# First genome sequences of *Achromobacter* phages reveal new members of the N4 family

**DOI:** 10.1186/1743-422X-11-14

**Published:** 2014-01-27

**Authors:** Johannes Wittmann, Brigitte Dreiseikelmann, Manfred Rohde, Jan P Meier-Kolthoff, Boyke Bunk, Christine Rohde

**Affiliations:** 1 Department of Microorganisms, Leibniz Institute DSMZ – German Collection of Microorganisms and Cell Cultures, Braunschweig, Germany; 2 Department of Microbiology/Genetechnology, Faculty of Biology, University of Bielefeld, Bielefeld, Germany; 3 Helmholtz Centre for Infection Research, Department of Medical Microbiology, Central Facility for Microscopy, Braunschweig, Germany; 4 Bioinformatics, Leibniz Institute DSMZ – German Collection of Microorganisms and Cell Cultures, Braunschweig, Germany

**Keywords:** *Achromobacter xylosoxidans*, N4-like phage, Genome, Lar-like protein, N4likevirus, Podoviridae, GBDP

## Abstract

**Background:**

Multi-resistant *Achromobacter xylosoxidans* has been recognized as an emerging pathogen causing nosocomially acquired infections during the last years. Phages as natural opponents could be an alternative to fight such infections. Bacteriophages against this opportunistic pathogen were isolated in a recent study. This study shows a molecular analysis of two podoviruses and reveals first insights into the genomic structure of *Achromobacter* phages so far.

**Methods:**

Growth curve experiments and adsorption kinetics were performed for both phages. Adsorption and propagation in cells were visualized by electron microscopy. Both phage genomes were sequenced with the PacBio RS II system based on single molecule, real-time (SMRT) technology and annotated with several bioinformatic tools. To further elucidate the evolutionary relationships between the phage genomes, a phylogenomic analysis was conducted using the genome Blast Distance Phylogeny approach (GBDP).

**Results:**

In this study, we present the first detailed analysis of genome sequences of two *Achromobacter* phages so far. Phages JWAlpha and JWDelta were isolated from two different waste water treatment plants in Germany. Both phages belong to the *Podoviridae* and contain linear, double-stranded DNA with a length of 72329 bp and 73659 bp, respectively. 92 and 89 putative open reading frames were identified for JWAlpha and JWDelta, respectively, by bioinformatic analysis with several tools. The genomes have nearly the same organization and could be divided into different clusters for transcription, replication, host interaction, head and tail structure and lysis. Detailed annotation via protein comparisons with BLASTP revealed strong similarities to N4-like phages.

**Conclusions:**

Analysis of the genomes of *Achromobacter* phages JWAlpha and JWDelta and comparisons of different gene clusters with other phages revealed that they might be strongly related to other N4-like phages, especially of the *Escherichia* group. Although all these phages show a highly conserved genomic structure and partially strong similarities at the amino acid level, some differences could be identified. Those differences, e.g. the existence of specific genes for replication or host interaction in some N4-like phages, seem to be interesting targets for further examination of function and specific mechanisms, which might enlighten the mechanism of phage establishment in the host cell after infection.

## Background

During the last years, the number of nosocomially acquired infections caused by multi-resistant *Achromobacter* strains has rapidly increased and become clinically relevant. *Achromobacter xylosoxidans* is a motile Gram-negative rod [1] that apart from being widely distributed in natural environments, mainly in soil [2] or different water sources [3], has been recognized as an emerging nosocomial pathogen potentially causing different human infections, including endocarditis [4,5], bacteremia [6,7], meningitis [8], ocular infections [9,10] or urinary tract infections [11]. Recent studies on antibiotic resistances in *Achromobacter* strains [12] showed a high number of resistances against antimicrobial substances from different classification groups, such as penicillins, lincosamides or cephalosporines. Other reports on beta-lactamases [13,14] and resistances against aminoglycosides [15] in *A. xylosoxidans* also stress the urgent need to search for new alternatives to fight this opportunistic pathogen. Bacteriophages as natural opponents of bacteria have become an interesting option for treatment of microbial infections in plants, animals and also humans. However, phages against *Achromobacter* species were nearly unknown, except for some old publications [16,17]. Recently, the study by Wittmann *et al*. [12] showed that bacteriophages against *Achromobacter* species can be easily found in the environment as a diverse set of different phages against *Achromobacter* could be isolated from soil and sewage samples. The majority of these bacteriophages belong to the *Siphoviridae* with long and flexible tails, but two members of the *Podoviridae*, phages JWAlpha and JWDelta, could be identified. Both are lytic phages that contain double-stranded DNA genomes, which are very similar, but not identical. Here we present the first genomic data on *Achromobacter* phages so far and further physiological analysis of both N4-like phages. *Escherichia* phage N4 was known as a genetic orphan for a long time [18], having no similarities to any other phages in regard to its conserved genomic structure that includes a large virion-encapsulated RNA polymerase which plays an important role in its transcription mechanism with three different RNA polymerases. Due to the increasing number of sequenced phage genomes, we now have numerous N4-like phages for members of the γ-Proteobacteria [19-21] and α-Proteobacteria [18]. In this study we present the first phages of the N4-like genus from *Achromobacter*, a member of the β-Proteobacteria.

## Results and discussion

### General phage characteristics

#### Morphology

**Figure 1:**
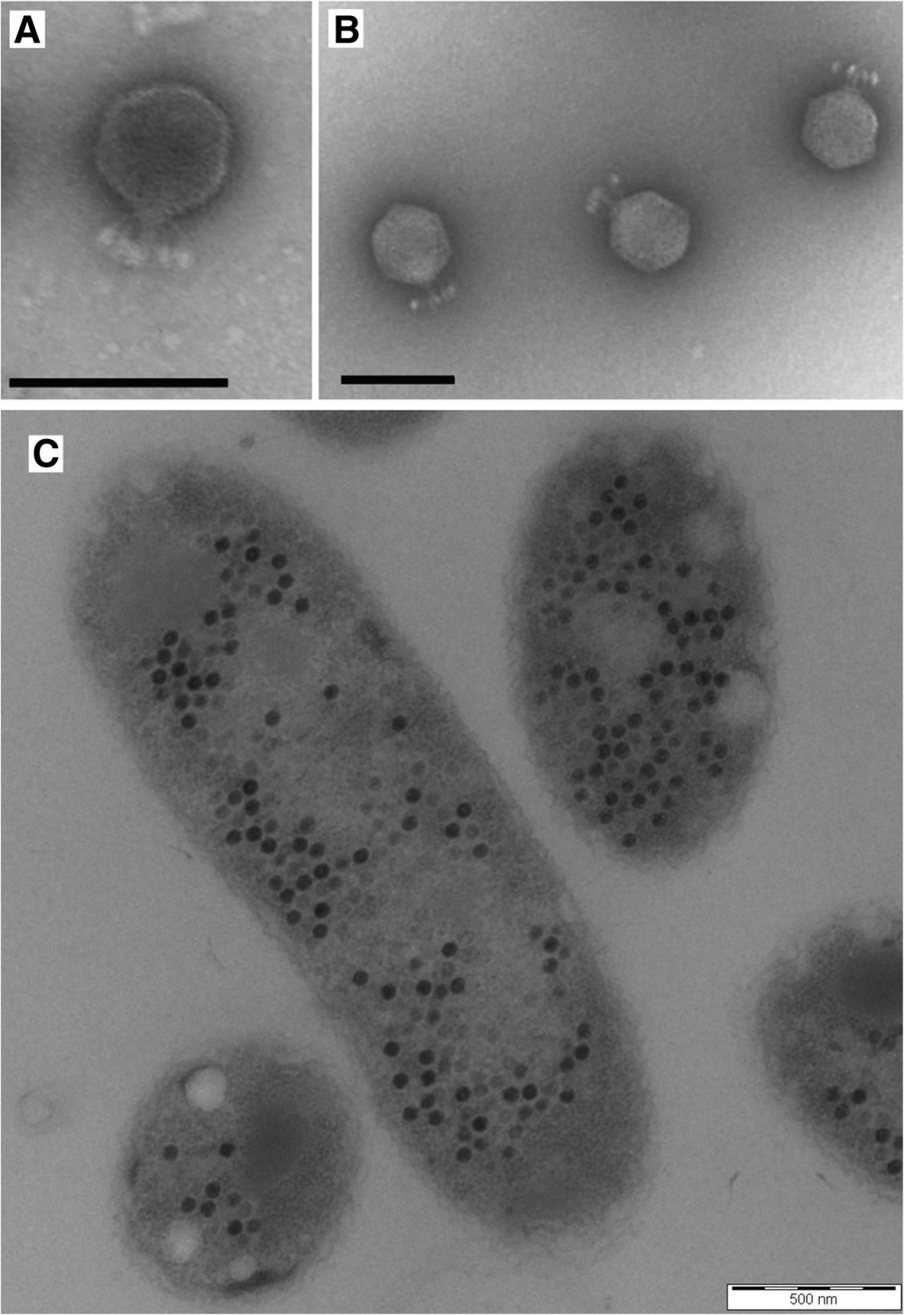
**Transmission electron micrographs of JWAlpha and JWDelta (A and B) and of an ultrathin section of *A. xylosoxidans *DSM 11852 cells, 90 min after infection with phage JWAlpha (C).** Scale bars represent 100 nm.

*Achromobacter* phages JWAlpha and JWDelta were isolated from waste water treatment plants in Werl and Braunschweig, respectively [12]. Morphological analysis by transmission electron microscopy revealed that both phages belong to the *Podoviridae* and seem to be quite similar. JWAlpha consists of an icosahedral head with a diameter of about 59 nm, whereas the head of JWDelta with a length of about 72 nm and a width of 67 nm is slightly longer. Both phages possess a short tail with an approximate length of 22 nm and several short tail fibers (Figure 1A and B).

### Adsorption parameters

**Figure 2:**
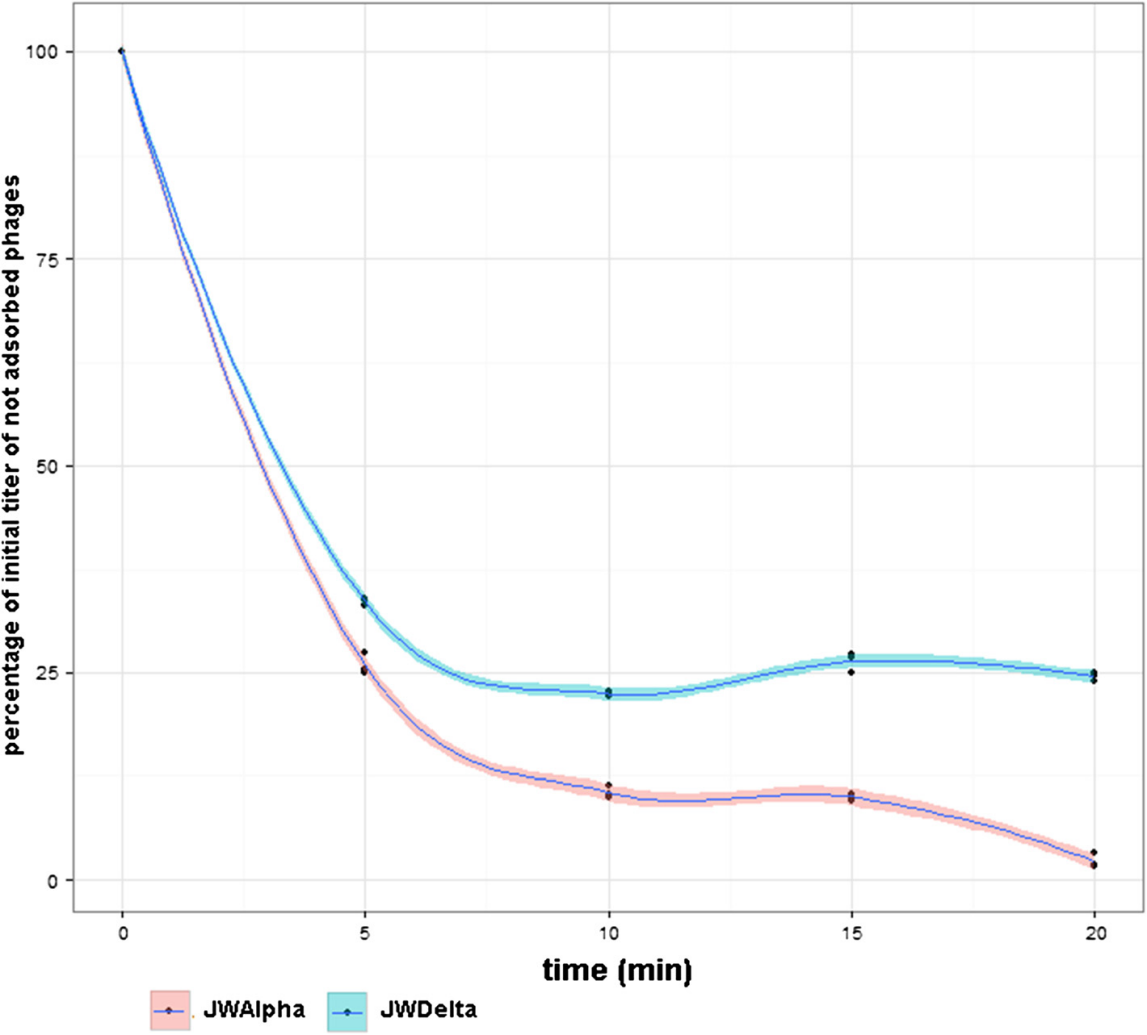
**Adsorption kinetics of JWAlpha and JWDelta to *Achromobacter *host cells (DSM 11852).** Bacterial cultures were infected with a multiplicity of infection of 0.1. Samples were taken at different time points to determine adsorption kinetics. Filtrates were diluted to determine titer of free phage particles in the sample. This experiment was performed in triplicate.

**Figure 3:**
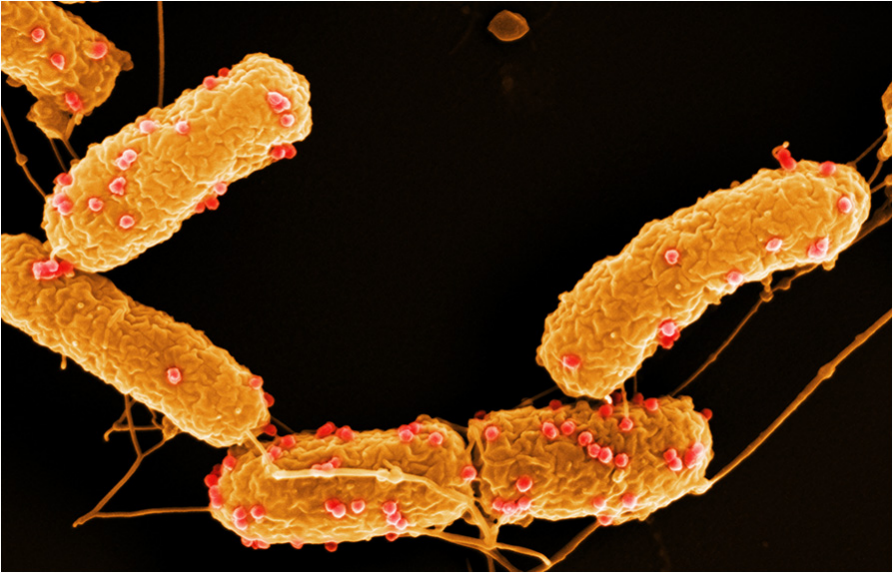
**Adsorption of phage JWAlpha to *Achromobacter *DSM 11852 cells.** (Scanning electron micrograph). Sample was taken 5 min after phage application. Adsorbed phages are coloured in red.

Experiments investigating the adsorption kinetics of the two phages showed that 5 min after adding phages to the bacterial host, about 65-70% of the phages have already adsorbed to their host cells (Figure 2). Whereas only about 1% of the originally applied JWAlpha phages were still detectable after 20 min, nearly 25% of the applied JWDelta phages could still be detected. According to the practical method presented by Kropinski [22], adsorption rate constant *k* was calculated for JWAlpha (*k* = 1.91 × 10^-9^ ml/min) and JWDelta (*k* = 1.56 × 10^-9^ ml/min). Scanning electron micrographs showed that adsorption of the phages takes place all around the cell surface (Figure 3). Further analyses revealed that no divalent cations like Mg^2+^ or Ca^2+^ were necessary for successful adsorption and penetration, as for example it was shown for phages PL-1 [23] or T5 [24] (data not shown).

### Burst size

**Figure 4:**
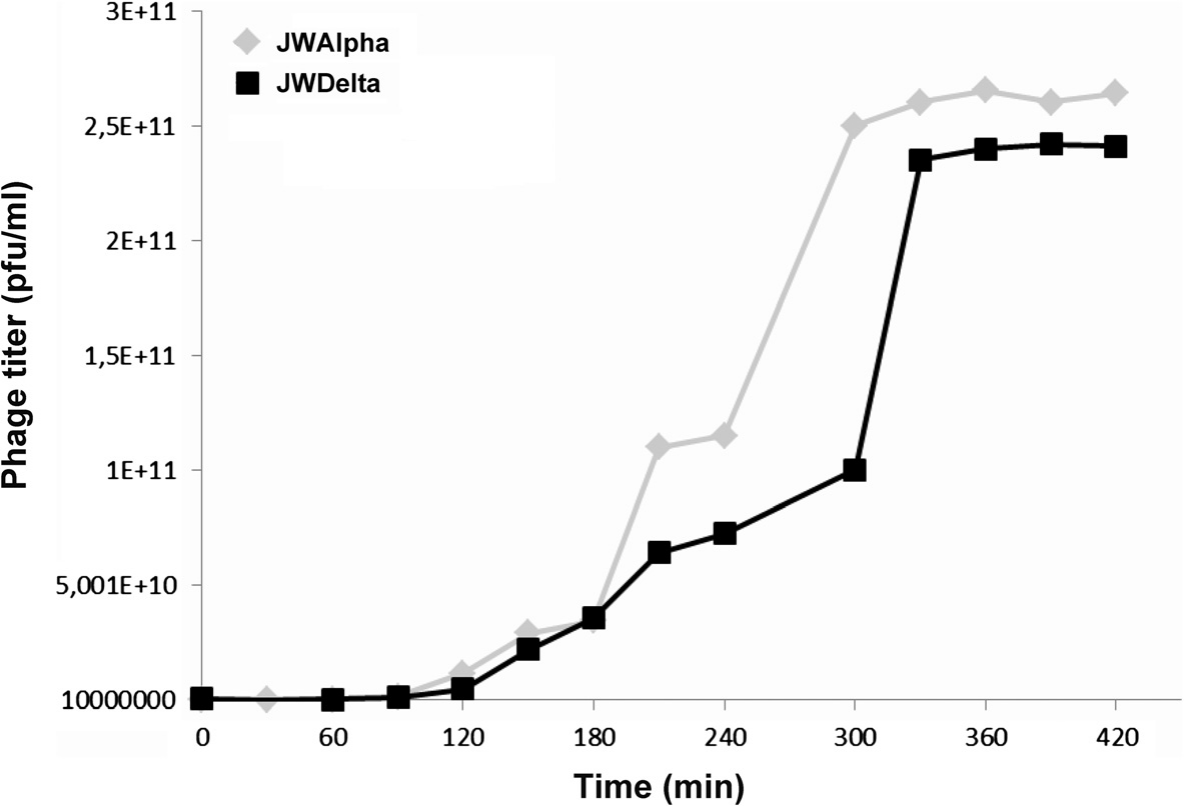
**One-step growth curve experiments with JWAlpha and JWDelta.** DSM 11852 cells were infected with a multiplicity of infection of 0.1.

In general, both phages form clear plaques (1–2 mm diameter) on bacterial lawns and lysates of both phages reveal high titers (~10^11^ pfu/ml). Electron micrographs of ultrathin sections of *Achromobacter* cells infected with both phages revealed a burst size of about 200 new phages per infected cell (Figure 1C). One-step growth curve experiments showed that both phages have latent periods about 1.5-2 h, after which an increasing number of free phages could be detected (Figure 4). Both curve plateaus with a titer of about 2×10^11^ pfu per ml were reached after 5 h. The determined burst size of about 180 correlates with the TEM ultra-thin section analysis.

### Stability

Incubation at different pHs ranging from pH 4-pH 10 for 1 hour and heating at different temperatures (30–60°C) for 10 min did not have any effect on the titer (data not shown) Storage of lysates over months at 4°C did not result in loss of titer, the phages seem to be stable.

### Genomics

First endonuclease restriction digestions of their DNA gave a first hint that they are not identical [12]. JWAlpha and JWDelta both contain linear, double-stranded DNA genomes with a length of 72329 bp and 73659 bp, respectively, comparable to all N4-like phages known so far. Both genomes reveal terminal redundant ends of 365 bp for JWAlpha and 420 bp for JWDelta. After analysis of the genomic DNA by pulsed field gel electrophoresis only one single band, but no concatemers could be identified (data not shown) which suggests that both genomes possess no cohesive ends as also previously described for phage N4 [25,26]. Additionally, a time-limited BAL 31 digestion and a following hydrolysis with restriction endonuclease SmaI revealed that the genomes are not circularly permutated since two distinct bands as representatives of the ends of the genomes were truncated (data not shown). The G + C contents of JWAlpha and JWDelta are 54.4% and 54.2%, respectively. In comparison with complete *Achromobacter* genomes sequenced so far (65-66% GC content) [27,28], the G + C contents of the phage genomes are significantly lower than those of possible hosts, which also occurs in other N4-like phage genomes [18]. Open reading frames (ORFs) were determined using MyRAST [29] and ARTEMIS based on three different start codons (ATG, GTG and TTG) and putative Shine Dalgarno sequences upstream the coding regions. Thus, we identified 92 ORFs for JWAlpha (88 ATG, 3 GTG and 1 TTG) and 89 ORFs for JWDelta (86 ATG, 2 GTG and 1 TTG), resulting in a coding percentage of 95.9% and 94.1%, respectively. Analysis of the genome sequences using tRNAscan-SE [30] did not reveal any genes for tRNAs, which leads to the assumption that both phages are adapted ideally to their host organism in regard to codon usage and do not require additional tRNAs of their own. Comparing the number of encoded tRNAs among all N4-like phages, it could be shown that most N4-like phage harbor between 1–3 genes for different additional tRNAs with codons that the host cell does not provide. Whereas *Sulfitobacter* phage pCB2047-B (Genbank accession number HQ317387, unpublished) and N4-like phages from *Salmonella* harbor 10 or more genes for different tRNAs, the genomes of the N4-like phages from the *Pseudomonas* group did not reveal any tRNA genes. Based on a work of de Paepe *et al.*, Bailly-Bechet *et al.* suggested that virulent phages have more tRNA genes than temperate phages to ensure optimal translation and therefore might replicate faster [31,32]. In this context, it would be interesting to compare the number of tRNAs with latency times of all N4-like phages.

**Figure 5:**
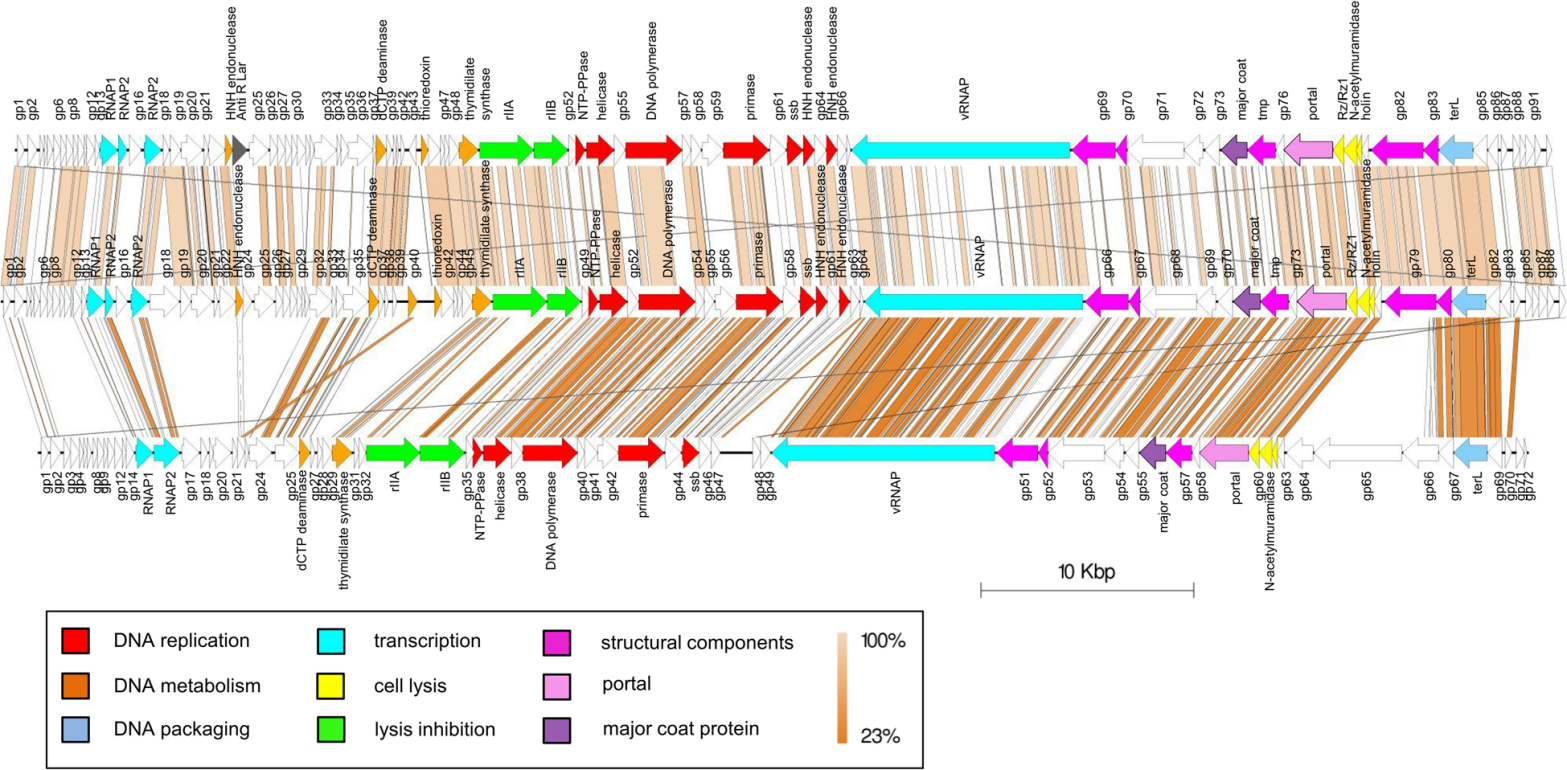
**Genome structures of phages JWAlpha and JWDelta and comparison with *Escherichia *phage N4.** Proposed functional clusters are marked by the same colour. Figure was generated using Easyfig with amino acid sequence comparison [33]. Level of amino acid identity range is shown via the gradient scale.

Though the two phages were isolated from different waste water treatment plants 250 km apart, they reveal identities of 98% at the nucleotide level and their complete genomic structure seems to be highly conserved. In general, the two phages share 87 genes with an amino acid identity range from 67 up to 100%, including 24 identical genes. BLASTP analysis identified 49 genes with strong similarities to phage N4 with an identity range between 25 and 84% at the amino acid level. Overall, compared to other members of the N4 family most similarities could be determined in phages from *Escherichia* (42–49 genes). Fewer genes with similarities to JWAlpha and JWDelta could be identified, for instance, in *Erwinia* phage vB_EamP-S6 (31), *Pseudomonas* phages (25–28) or *Vibrio* phages (20–21). Both genomes show nearly the same organization with two large gene clusters located on different strands and a small cluster at the end of the genome that is transcribed towards a different direction again (Figure 5). These large clusters can be divided into different subclusters for so-called early genes, DNA metabolism, host interaction, transcription, replication, structural genes, lysis and DNA packaging.

### Genes for transcription

*E. coli* phage N4 has been recognized to be a genetic orphan for a long time, being the only known phage that did not depend on the RNA polymerase (RNAP) of its host to transcribe its early genes. N4-like phages that have been described before harbor at least three genes for RNAPs for the transcription of genes in different stages of their life-cycle [34]. The most striking and highly conserved characteristic of a N4-like phage is the gene for a large RNAP with around 3500 amino acids that is encapsulated in the virion and is injected into the host cell together with the phage DNA for immediate start of early gene transcription [35,36]. Both *Achromobacter* phages also harbor three different RNAPs, suggesting the same transcription organization as N4 and its other relatives. The large virion RNAPs consist of 3425 amino acids in both phages, only differing in 10 amino acid residues and containing no cysteine residues as also reported by Kazmierczak et al. [37]. Cysteine residues in a protein might form disulfide bonds and lead to a change of protein folding and tertiary structure, which would make it difficult or even impossible for such a large protein to be injected through the phage tail into the host cell. Like other N4-like phages JWAlpha and JWDelta also carry genes that encode two different RNA polymerase subunits, RNAP1 and RNAP2, which play a role in the transcription of N4 middle genes [38]. Similar to phage N4 the gene for RNAP1 is directly followed by RNAP2, whereas other N4-like phage genomes show insertions of small genes in this area [18,20,39]. In contrast to other N4-like phages the gene for RNAP2 is interrupted by a gene similar to gp8 of *Celeribacter* phage P12053L in both *Achromobacter* phages, dividing it into two parts and raising the question whether it is still functional.

### Gene products involved in host interaction

The so-called early genes of a phage genome often encode proteins that somehow interact with the host, for example to protect the newly-injected phage DNA molecule from degradation in the host cytoplasma and see to its establishment, to shut off the host metabolism or to modify host polymerases for transcription in favor of phage propagation. Phages can shut off their host metabolism in different ways, e.g. by host DNA degradation [40,41]. Lavigne et al. [42] recently identified gp13 of *Pseudomonas* phage LUZ19 as an acetyltransferase that hinders both replication and transcription in *Pseudomonas* posttranslationally by acetylation. In regard to DNA protection, for example, phages use different strategies to avoid degradation by host nucleases. Phage T4 e.g. protects its free genomic ends from degradation by exonucleases with the help of small DNA-binding proteins [43], phage T7 possesses an anti-restriction system that inhibits the activity of the bacterial restriction endonuclease by prevention of its binding to the phage DNA [44,45]. Phage JWAlpha also harbors a gene (gp24) that might play a role in a putative anti-restriction system. Its deduced amino acid sequence reveals a conserved domain with homologies to restriction alleviation protein Lar from the defective prophage Rac [46] which is able to alleviate restriction and to enhance modification by the restriction and modification system in *E. coli* at the same time. This is apparently accomplished by altering the activity of a type I methyltransferase to efficiently methylate the phage DNA and protecting it against degradation [47]. Though the two phages have a quite similar genome organization, we could not identify this gene in JWDelta. The deduced amino acid sequence of a putative candidate at the same genomic position as in JWAlpha was comparatively much shorter and did not reveal any homologies to proteins of JWAlpha or Lar-like proteins from the database. Whether JWDelta just lacks this gene or whether the gene product of this putative candidate has a similar function, but a yet unknown structure stays to be examined. In general, the different mechanisms of host takeover after infection are worth closer examination in the future.

Another interesting example for a host interaction system could also be found in both *Achromobacter* phage genomes. Upstream the replication cluster two genes were assigned that share 99% and 98% identities at the nucleotide level with the homologous gene in the other phage, respectively. Their deduced amino acid sequences reveal homologs to a rIIA-like and a rIIB-like protein from phage N4. This kind of proteins were first described in phage T4 and might play a role in lysis inhibition among other functions that are still not completely clear, but they seem to have an influence on cell energetics, including Mg^2+^ transport and ATP biosynthesis [48]. Examining all N4-like phage genomes known so far for these genes, we discovered that homologs of both genes are widely distributed among N4-like genomes in different host genera. They could also be identified in N4-like phage genomes from the *Roseobacter* clade, from *E. coli* and *Pseudomonas*, but neither in *Erwinia* phage vB_EamP_S6 nor in *Salmonella* or *Vibrio* phages. Generally, both phage genomes are quite similar in their structure and number of genes. The only differences between them could be found in the area between the early genes for RNA polymerases and the genes for rIIa- and rIIB-like proteins. Apart from the already mentioned gene for a Lar-like protein, JWDelta lacks homologous genes for *orf29*, *orf37* and *orf41* from JWAlpha. In comparison with JWDelta, the genome of JWAlpha showed two deletions which resulted in a much shorter *orf18* with an unclear function and a missing gap of about 1,3 kb that contains a gene for a HNH endonuclease in JWDelta.

### Replication cluster and genes for nucleotide metabolism

Both phage genomes of this study harbor genes for a complete replication cluster in the same order as N4 and other N4-like phages, including a gene for a nucleoside triphosphate pyrophosphohydrolase (Superfamily cl16941) that might provide the helicase with energy by hydrolysis of NTPs, and a gene for a DNA helicase containing a conserved domain UvrD_C_2 (pfam13538) which resembles a AAA-like structural fold that is often found at the C-terminus of helicases. Moreover, this cluster contains a gene that codes for a DNA polymerase with a 3'-5' exonuclease proofreading domain (35EXOc (smart00474)) and a second conserved domain at the C-terminus, DNA_pol_A (pfam00476). The N4 gp43-like gene for a primase was identified based on its similarities to other primase genes from several phages at the amino acid level and the C-terminal Primase C domain PriCT_1 (pfam08708). At the end of the replication cluster we assigned a gene for a putative single-stranded binding protein (Ssb) that though lacking a conserved domain showed a characteristic glycine- and proline-rich C-terminus. The *ssb* gene is followed by two genes for putative endonucleases, their deduced amino acid sequences revealed characteristic conserved domains NUMOD4 (pfam07463) and HNH_3 (pfam13392) for putative HNH homing endonucleases. Both phages also possess genes involved in the DNA metabolism, to be specific genes for a deoxycytidine triphosphate deaminase with a conserved dcd (PRK00416) domain, a thymidylate synthase (Thy1 (pfam02511)), a homing endonuclease (HNH_3 (pfam13392)) and a thioredoxin with a conserved TRX family domain (cd02947) that might play a role in the regulation of some enzymes involved in DNA metabolism or replication. In contrast to N4-like phages from the *Roseobacter* clade that are the only other members that also carry genes for thioredoxin, the *Achromobacter* phages do not harbor a gene for a ribonucleotide-diphosphate reductase, an enzyme involved in formation of deoxyribonucleotides. In general, the genes of the replication cluster are highly conserved and show strong similarities to genes from other N4-like phages at the amino acid level, e.g. the genes for the helicase, DNA polymerase or Ssb. However, besides these genes, there are other genes (*orf*52, *orf*55, *orf*57 and *orf*58) with a yet unclear function that reveal similarities only to N4-like phages from *Escherichia* and *Erwinia*.

### DNA packaging proteins and structural proteins

**Figure 6:**
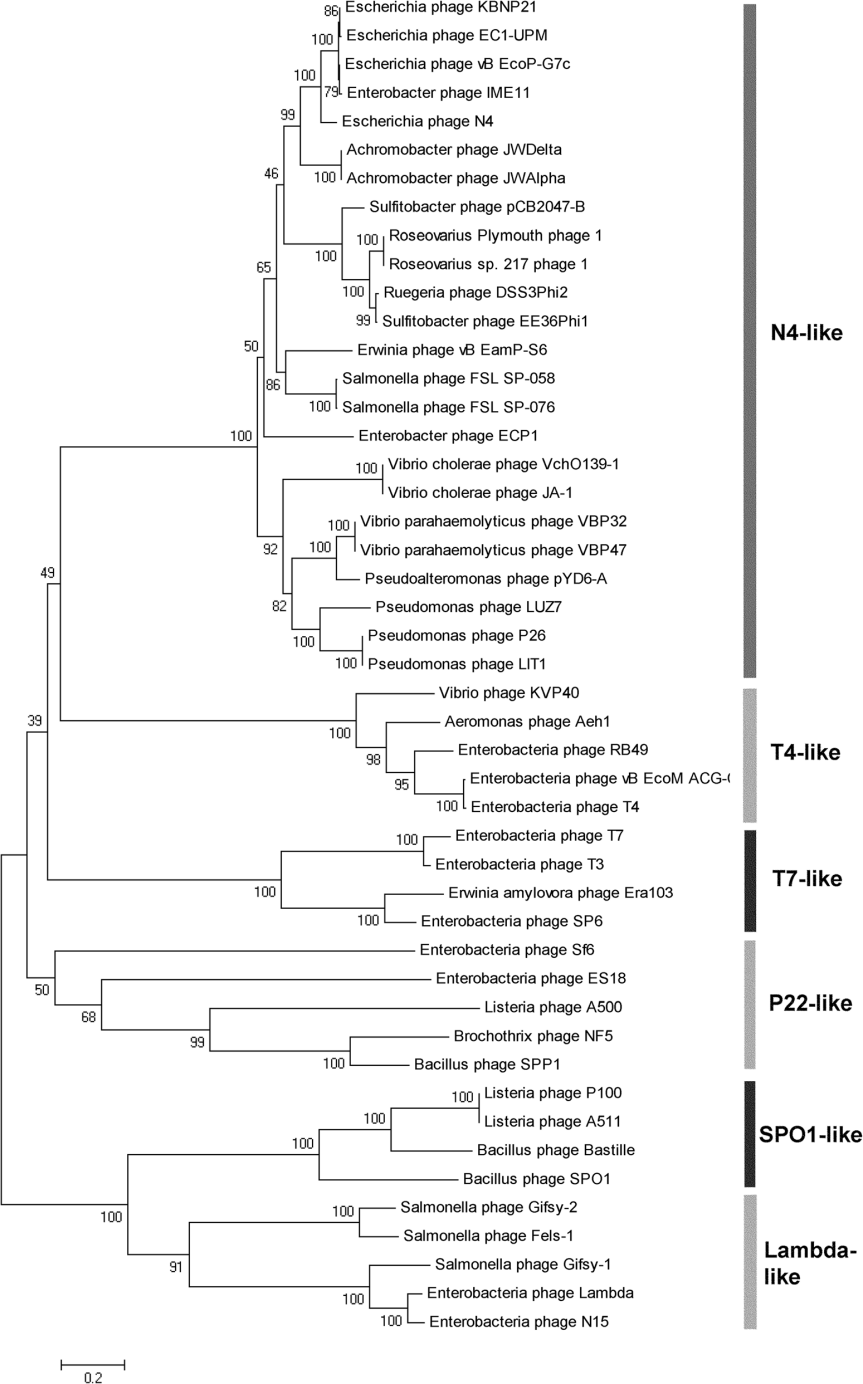
**Phylogenetic analysis of terminase large subunits of JWAlpha and JWDelta compared to other N4-like phages and phages with other known DNA packaging strategies.** Neighbor-joining tree was constructed based on ClustalW alignment of terminase subunit amino acid sequences with 1000 bootstrap replicates (MEGA5).

In general, phages have developed different strategies for packaging DNA into their capsids, depending on their mechanism of replication, head size and its subsequent ability to hold a little bit more than one complete phage genome, which often leads to circularly permutated genomes. For instance, in phage T4 the head full of DNA is about 3% longer than the genome size [49]. Usually, two main proteins are used for DNA packaging, called terminases. These proteins display a hetero-multimeric structure with a small subunit for DNA binding and a large subunit with an ATPase domain and an endonuclease function. In most phage genomes, the terminase genes can be identified upstream the cluster for structural genes. In our study, the genes for the large terminase subunits could be clearly identified in both *Achromobacter* phage genomes. A phylogenetic analysis with other N4-like phages and several phages from a reduced set used by Fouts et al. [21] with different known DNA packaging strategies using a neighbor-joining method revealed that the *Achromobacter* terminases cluster together with all other terminases from N4-like phages and probably use the same mechanism for packaging based on direct repeats (Figure 6).

**Figure 7:**
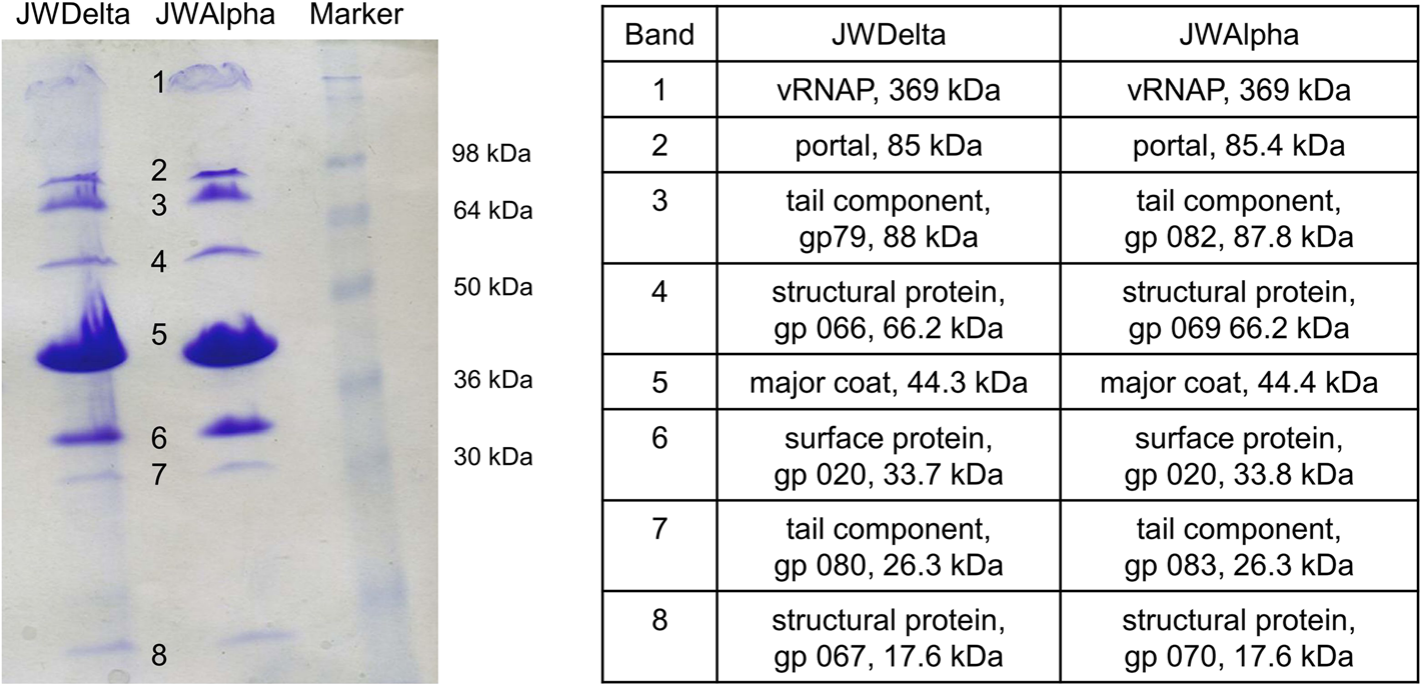
**Identification of structural proteins of phages JWAlpha and JWDelta by SDS-PAGE analysis.** Protein profiles of JWDelta and JWAlpha. Proteins were separated on a 17.5% SDS-PAGE. After extraction of gel slices from corresponding bands and tryptic digestion, samples were analyzed via peptide mass fingerprinting. Proteins identified by peptide mass fingerprinting analysis are listed aside along with their approximate molecular weight.

After separation of denatured phage particles by SDS-PAGE, the protein profiles of JWAlpha and JWDelta were not distinguishable (Figure 7). Both profiles displayed eight distinct protein bands with nearly the same molecular weight that were further assigned by peptide mass fingerprinting, all bands corresponded to coding sequences from our DNA annotation. In bothprotein profiles, bands with approximately the same molecular weight also correspond to homologous genes with the same function. Peptides from the most prominent band (band 5) in both phages matched to a predicted major coat protein with a molecular mass of about 44 kDa that appears to make the most important structural protein of the phage head. With one exception (band 6), all examined protein bands correspond to deduced amino acid sequences of genes that were identified on the complement strand of the genome and encode structural proteins, such as a putative portal protein (band 2) or putative tail proteins (bands 3 and 7). Protein band 6 is the only example that is encoded by a gene that is not located in the gene cluster for structural proteins. Nevertheless its deduced amino acid sequence revealed a conserved bacterial Ig-like domain (Big_2 [pfam02368]) that occurs in phage surface proteins and might decorate the phage capsid after assembly. Besides all these structural proteins, one non-structural protein could be assigned by peptide mass fingerprinting. The largest band of both profiles (band 1) corresponds to the annotated RNA polymerase with an approximate molecular weight of about 369 kDa in both phages. As it is encapsulated in the virion to be injected into the host cell together with the phage genome, it was no surprise to detect this protein after complete denaturation of the phage capsid.

**Figure 8:**
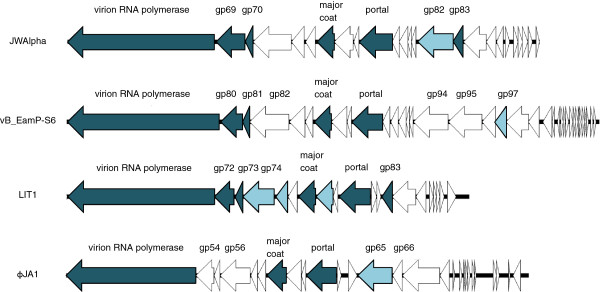
**Comparison of structural gene clusters of different N4-like phages displaying homologous genes identified by peptide mass fingerprinting.** All genes upstream the gene for the virion RNA polymerase are shown. Homologous genes for proteins that could also be identified by peptide mass fingerprinting in other examined phages are marked in dark blue, genes for proteins that could be identified just in one phage are marked in pale blue.

Analysis and comparison of protein profiles from different N4-like phages [20,21,39] with JWAlpha and JWDelta showed that in all cases the most prominent protein band was the major head protein with a molecular mass of about 44 kDa. Despite their similar morphology, the protein profiles of these phages differ in number and molecular mass of prominent bands. However, there is a series of ten genes starting with the gene for the virion RNA polymerase and ending with the gene for the portal protein that can be determined in the same order in other N4-like phages (Figure 8). In general, based on the area upstream of this conserved gene cluster, the N4-like phages can be divided into three different groups. The *Achromobacter* phages JWAlpha and JWDelta, phage N4 and other N4-like *Escherichia* phages, *Erwinia* phage vB_EamP-S6 and N4-like phages from the *Roseobacter* clade reveal genes of the lysis cluster upstream the gene for the portal protein. In case of the N4-like *Pseudomonas* and *Salmonella* phages and the *Vibrio* phages vB_VchP_VchO139-I and vB_VchP_JA1, the described cluster is preceded by genes which are transcribed towards a different direction. In *Vibrio* phages VBP32 and VBP47 the cluster consists of 11 genes and shows differences in regard to the length of some genes. Most of the structural genes are highly conserved, e.g. the genes for the major coat protein or the portal protein (Table 1). Some genes of JWAlpha and JWDelta, however, reveal only similarities to genes from N4-like phages from *Escherichia*, as described above for genes of the replication cluster.

**Table 1:** Comparison of homologous structural genes of different N4-like phages

**Identified proteins of JWAlpha**	**Homologous genes in *Erwinia *** **phage vB_EamP-S6**	**Homologous genes in *Pseudomonas *** **phage LIT1**	**Homologous genes in *Vibrio *** **phage φJA1**
vRNAP, 369 kDa	gp79, 380.6 kDa, 28% aa identities	gp71, 369.7 kDa, 23% aa identities	gp53, 335 kDa, 23% aa identities
Structural protein, gp69 66.2 kDa	gp80, 55.9 kDa, no similarities at the aa level	gp72, 52.5 kDa, no similarities at the aa level	gp54, 47.1 kDa, no similarities at the aa level, not identified
Structural protein, gp70, 17.6 kDa	gp81, 16.3 kDa, 40% aa identities	gp73, 16.6 kDa, 38% aa identities	gp55, 15.3 kDa, 31% aa identities, not identified
Major coat, 44.4 kDa	gp85, 43.7 kDa, 66% aa identities	gp77, 44 kDa, 53% aa identities	gp59, 47 kDa, 44% aa identities
Portal, 85.4 kDa	gp88, 81.3 kDa, 60% aa identities	gp80, 81.7 kDa, 48% aa identities	gp62, 79.2 kDa, 47% aa identities
Tail component, gp82, 87.8 kDa			gp65, 87.3 kDa, no similarities at the aa level, not identified
Tail component, gp83, 26.3 kDa	gp97, 26.7 kDa, 36% aa identities, not identified	gp83, 27.9 kDa, 26% aa identities	gp66, 25.8 kDa, 32% aa identities, not identified
Surface protein, gp20, 33.8 kDa			

Orf, molecular mass and similarities to JWAlpha at the amino acid level are shown. If not identified by peptide mass fingerprinting, proposed homologs in the phage genomes are listed.

### Gene products for host cell lysis

After assembly of new phage progeny, most phages lyse their bacterial host cell in order to release the new phages into the environment. For that purpose, they often use a dual lysis system that consists of a holin that forms a pore in the cytoplasmic membrane to release the endolysin into the periplasmic space where it enzymatically degrades the peptidoglycan [50]. In general, due to the much higher complexity of the Gram-positive cell wall with a lot of different peptidoglycan subtypes [51], endolysins of phages with Gram-positive hosts possess a lot of highly specific enzymatic activities. However, most of the phages with Gram-negative hosts use unspecific N-acetylmuramidases for degradation of the peptidoglycan as there is just one peptidoglycan layer to overcome. The lysis clusters of both *Achromobacter* phages consist of three genes (*orf*78-*orf*80 in JWAlpha, *orf*75-*orf*77 in JWDelta) and show the same organization as lysis clusters from other phages with Gram-negative hosts. Analysis with TMHMM Server v. 2.0 [52] revealed that the deduced amino acid sequences of *orf80* from phage JWAlpha and *orf77* from JWDelta both contain two transmembrane regions and show similarities to putative holins of *Escherichia* phages EC1-UPM and vB_EcoP_G7C, hence both proteins could be assigned as class II holins [53]. The gene for the holin is followed by the endolysin gene, BLASTP analysis of the amino acid sequences identified them both as N-acetylmuramidases. Downstream the endolysin genes, one small ORF for a protein could be assigned that also revealed one transmembrane region and similarities to a Rz-like protein. Besides genes for a holin and an enzymatically active endolysin, phages of gram-negative hosts often harbor genes for proteins Rz and Rz1 that somehow interact with the outer membrane and play a role in the final step of host lysis [54,55]. Examining all N4-like genomes in regard to their lysis clusters, we could identify five different groups of clusters with apparently genus-specific strategies for host lysis. The largest group of the N4-like phages harbors genes for a class II holin with two transmembrane regions, a N-acetylmuramidase and a Rz protein with one transmembrane region as described above for both *Achromobacter* phages. Further members of this group are the *Escherichia* phages N4, EC1-UPM, KBNP21 and vB_EcoP_G7C, *Erwinia* phage vB_EamP_S6 and *Enterobacter* phage IME11. The second group comprises the N4-like phages from the *Roseobacter* clade and *Enterobacter* phage ECP1 which all encode genes for a lysis protein. Since none of their deduced amino acid sequences revealed homologies to muramidases or to peptidases of the MEROPS database, they were apparently annotated as lysis proteins with yet unclear functions. In all roseophage genomes of this group, this gene is framed by two small orfs with one deduced transmembrane region. Though the *Enterobacter* phage ECP1 also possesses a gene that encodes a lysis protein, the structure of its lysis cluster is different. The whole cluster is located on the other DNA strand and is not transcribed towards the same direction as the preceding and following genes. In addition to that the gene for the lysis protein is preceded by two genes for proteins with three transmembrane regions each, which is characteristic for class I holins. The N4-like *Salmonella* phages FSL SP-058 and FSL SP-076 belong to the third group with a different lysis cluster. Like phage ECP1, both *Salmonella* phages possess two putative genes for class I holins, but in addition to a lysis protein they also harbors a gene for a putative lysozyme and, thus, they are the only N4-like phages with two different enzymes for host lysis. The fourth group consists of three *Pseudomonas* phages of the N4 family, namely P26, LIT1 and LUZ7. Though two putative holin genes could be identified in all three genomes, no muralytic enzyme could be predicted. The endolysin containing region seems to be completely deleted [20] which raises the question whether there are other muralytic enzymes with so far unknown mechanisms. Genomes of members of the fifth group, mainly *Vibrio* phages, even do not reveal any sign of lysis cluster at all which makes them interesting candidates for examining their lysis mechanism and potentially identifying new muralytic enzymes. Since all bacteriophages of the N4-family known so far have gram-negative hosts, it was no surprise that nearly all of them encode genes for rather unspecific muramidases and no specific endopeptidases as often found in phages with gram-positive hosts [56]. Although the genomes of N4-like phages are highly conserved both in sequence identity and structure, it is interesting to observe these differences in their lysis clusters. In contrast to the replication cluster or the cluster for structural genes, a broader diversity could be determined which is worth further examination.

### Phylogenomic analysis

**Figure 9:**
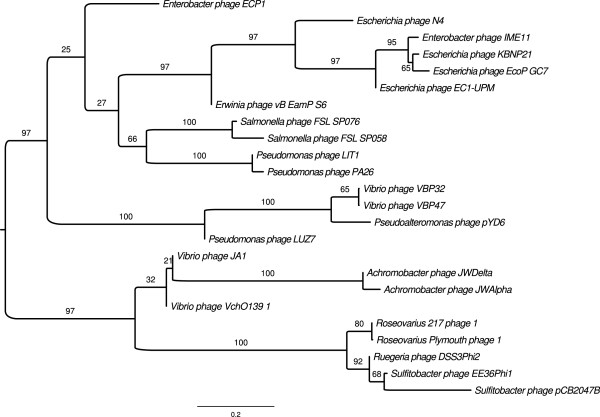
**Phylogenetic tree based on the proteomes of 24 N4-like phages.** The tree was inferred from pairwise distances, assessed via the GBDP approach [57-59], and using the balanced minimum evolution principle [60]. The tree was rooted via midpoint rooting [61]. GBDP used BLAST + [62] as a local alignment programme (i.e., blastp using an initial word length of 3) and applied the following settings for the pairwise distance calculation: trimming algorithm, formula d5 and no e-value filter.

Figure 9 shows the resulting proteome-based phylogenetic tree of the 24 phage genomes. The group of the newly discovered *Achromobacter* phages is part of a larger clade that contains phages associated to the family *Rhodobacteraceae* as well as *Vibrio* phages JA1 and VchO139-1. But whereas the clades related to the phages of *Rhodobacteraceae* as well as *Achromobacter* have both high support, the positioning of the denoted *Vibrio* phages is not supported. Overall, the tree yields the expected relationships of the different groups (e.g., as previously reported [21]) and, since it provides branch support, dubious positioning (usually due to the lack of data) can now be directly identified and discussed accordingly.

## Conclusions

Analysis of the genomes of *Achromobacter* phages JWAlpha and JWDelta and comparisons of different gene clusters with other phages revealed strong similarities to other N4-like phages, especially of the *Escherichia* group. Phages of the N4 family infect a lot of different genera with *Achromobacter* as a new member. Although all these phages show a highly conserved genomic structure and partially strong similarities at the amino acid level, interestingly, some differences could be identified probably depending on their host genus. These differences, e.g. the existence of a gene for a ribonucleotide-diphosphate reductase in N4-like *Roseobacter* phages or the completely different structure of the replication cluster in N4-like phages of *Pseudomonas* compared to all other N4-like phages, seem to be interesting targets for further examination of function and specific mechanisms. In particular, further examination of the gene for a Lar-like protein in JWAlpha might enlighten the mechanism of phage establishment in the host cell after infection. A closer look on the mechanisms for host lysis in those phages that revealed no or yet unknown enzymes for cell wall degradation might also be interesting for the identification of new muralytic enzymes.

## Material and methods

### Genome sequence accession numbers

Genome sequences of JWDelta and JWAlpha were deposited to Genbank under accession numbers KF787094 and KF787095, respectively. Further genome sequences of N4-like phages for comparison could be obtained under following accession numbers: *Enterobacteria* phage N4 (EF056009), *Escherichia* phage vB_EcoP_G7C (HQ259105) [63], *Escherichia phage* KBNP21 (JX415535) [64], *Enterobacter* phage EcP1 (HQ641380), *Enterobacter* phage IME11 (JX880034) [65], *Escherichia* phage EC1-UPM (KC206276) [66], *Erwinia* phage vB_EamP-S6 (HQ728266) [39], *Pseudoalteromonas* phage pYD6-A, *Pseudomonas* phage LIT1 (FN422399), *Pseudomonas* phage LUZ7 (FN422398) [20], *Pseudomonas* phage PA26 (JX194238) [67], *Roseovarius* sp. 217 phage 1 (FR682616), *Roseovarius* Plymouth Podovirus 1 (FR719956), Ruegeria phage DSS3φ2 (FJ591093), Sulfitobacter phage EE36φ1 (FJ591094) [18], *Sulfitobacter* phage pCB2047-B (HQ317387), *Salmonella* phage FSL SP-058 (KC139517), *Salmonella* phage FSL SP-076 (KC139520) [19], *Vibrio* phage VBP32 (HQ634196), *Vibrio* phage VBP47 (HQ634194), *Vibrio* phage JA-1 (KC438282), *Vibrio* phage VCO139 (KC438283) [21].

### Bacterial strains and growth

*Achromobacter xylosoxidans* strain DSM 11852 was used for phage propagation and adsorption tests. It was cultured in liquid tryptic soy broth TSB (30 g^-l^, pH 7.5, OXOID) medium or on Petri dishes of TSB medium supplemented with 1.5% agar (w/v) for approximately 16 h (overnight) at 28°C.

### Purification of phages and phage DNA

Purification of the phages by CsCl gradient centrifugation and of their DNA by phenol extraction was done as previously described [68].

### Sample preparation and sequencing

SMRTbell™ template libraries were prepared according to the instructions from Pacific Biosciences, Menlo Park, CA, USA, following the Procedure & Checklist for “Low-Input 10 kb Template Preparation and Sequencing” using C2-C2 Chemistry.

Briefly, for preparation of 10 kb libraries ~15 μg genomic DNA was sheared in an Eppendorf microcentrifuge 5424 2×3 min at 5500 rpm using g-tubes™ from Covaris, Woburn, MA, USA. Size range was monitored on an Agilent 2100 Bioanalyzer from Agilent Technologies, Santa Clara, USA. DNAs were concentrated, end-repaired and ligated to hairpin adapters applying components from the DNA/Polymerase Binding Kit 2.0 from Pacific Biosciences, Menlo Park, CA, USA. Reactions were carried out according to the manufacturer´s instructions with exception of the ligation, which took place 2 h at 25°C and overnight at 4°C. SMRTbell™ templates were exonuclease treated for removal of incompletely formed reaction products. A mixture of Exonuclease III and Exonuclease VII (Affymetrix, High Wycombe, UK) was utilized. Hereby, an additional exonuclease treatment after the second purification took place, followed by three purification steps as recommended by Pacific Biosciences. Conditions for annealing of sequencing primers and binding of polymerase to purified SMRTbell™ templates were assessed with the Calculator in RS Remote, PacificBiosciences, Menlo Park, CA, USA. SMRT sequencing was carried out on the PacBio RS (PacificBiosciences, Menlo Park, CA, USA) using the DNA Sequencing Reagent 2.0. For each 10 kb libraries one 120 minutes movie was taken.

### Bioinformatics analyses

Phage genome assemblies were performed using the “RS_Preassembler_Allora.1” protocol included in SMRTPortal version 1.3.3. Within that following parameters were applied. For Filtering: Minimum Readlength 100, Minimum Subreadlength 500, Minimum Read Quality 0.8. For Assembly: Minimum Seed Read Length: 3500 for phage JWAlpha, BLASR Options (Advanced) minReadLength 200 maxScore 1000 bestn 24 maxLCPLength 16 nCandidates 24, Trim FASTQ Output true, Use CCS false, Overlap Permissiveness Least permissive, Expected Genome Size 50000, Maximum iterations 10, Detect Chimeras true. In both cases one final contig could be obtained. A quality check of that given consensus sequences regarding overall coverage as well as SNPs was performed using SMRT View and IGV [69] after application of the “RS_Resequencing.1” protocol with default parameters. The phage genomes were annotated using RAST [29] with subsequent manual curation in Artemis [70].

### Phage adsorption test and growth curve experiments

For adsorption assays, exponentially growing cultures of *Achromobacter xylosoxidans* strain DSM 11852 were infected with phages JWAlpha and JWDelta, respectively, with a multiplicity of infection (moi) of 0.01, for growth curve experiments, cells were infected with a moi of 0.1. Samples for both experiments were taken at different time points and directly filtered (membrane syringe filter 0.45 μm, Sartorius, order number 16555). Filtrates were diluted and spotted on a bacterial lawn of DSM 11852 to determine titer of free phages in the culture.

### SDS-PAGE analysis of phage proteins

Treatment of phage samples for SDS-PAGE analysis and SDS-PAGE analysis itself were performed as previously described [71].

### MALDI-TOF analysis

Coomassie stained protein bands were excised from the gel. The slices were washed twice in H_2_O for 5 min and dehydrated by adding 200 μl 50% acetonitrile for 5 min. Gel pieces were then washed in 200 μl 0.1 M NH_4_HCO_3_ for 15 min at room temperature. Washing solution was discarded and slices were dehydrated by adding acetonitrile again. Dehydrated gel pieces were completely dried in Speed Vac for 10 min. For the tryptic digestion of the proteins the gel slices were reswelled by adding 50 μl digestion solution containing trypsin and incubated over night at 37°C. For extraction of peptides 50 μl of acetonitrile were added, followed by incubation under vigorous shaking for 15 min at 37°C. Supernatants were removed and gel slices were treated with 50 μl 5% formic acid under vigorous shaking for 15 min at 37°C. After that 50 μl of acetonitrile were added, followed again by incubation under vigorous shaking for 15 min at 37°C. Supernatants were united with the removed supernatants and concentrated via Speed Vac. 40 μl 32% methanol/0.25% HCOOH were added, followed by 3 min treatment with ultrasound and analyzed by MALDI Ultraflex-TOF/TOF. Results were compared with a database of predicted phage proteins using Mascot. MALDI-TOF analysis was done at the Helmholtz Centre for Infection Research, Braunschweig, Germany.

### Negative staining of phages

Thin carbon support films were prepared by sublimation of a carbon thread onto a freshly cleaved mica surface. Phages were negatively stained with 0.5% (w/v) aqueous uranyl acetate, pH 5.0, according to the method of Valentine et al. [72]. Samples were examined in a TEM 910 transmission electron microscope (Carl Zeiss, Oberkochen) at an acceleration voltage of 80 kV.

### Field emission scanning electron microscopy (FESEM)

Bacteria incubated with phage JWAlpha were fixed with 2% glutaraldehyde and 5% formaldehyde, washed with TE-buffer (20 mM TRIS, 1 mM EDTA, pH 6.9) then dehydrated in a graded series of acetone (10, 30, 50, 70, 90, 100%) on ice for 15 min for each step, critical-point dried with liquid CO_2_ (CPD 30, Bal-Tec, Liechtenstein) and covered with a gold-palladium film by sputter coating (SCD 500, Bal-Tec, Liechtenstein) before being examinated in a field emission scanning electron microscope (Zeiss Merlin) using the Everhart Thornley SE detector and the inlens detector in a 25:75 ratio at an acceleration voltage of 5 kV.

### Transmission electron microscopy (TEM)

Samples were fixed in medium with 5% formaldehyde after 1 min 2% glutaraldehyde was added and kept on ice for 1 hour, washed with cacodylate buffer, and further fixed with 1% osmium in 0.08 M cacodylate buffer for 1 hour at room temperature. Then, samples were dehydrated with a graded series of acetone (10, 30, 50%) on ice. At the 70% dehydration step samples were incubated overnight in 70% acetone containing 2% uranyl acetate at 4°C. On the next day samples were further dehydrated with 90% and 100% acetone on ice, the 100% aceton step was repeated at room temperature. Samples were embedded in the epoxy resin Spurr according to described procedures [73]. Ultrathin sections were cut with a diamond knife, picked up with butvar-coated grids, counterstained with uranyl acetate (3 min) and lead citrate (15 sec), and examined in a TEM910 transmission electron microscope (Carl Zeiss, Oberkochen) at an acceleration voltage of 80 kV. Images were recorded digitally at calibrated magnifications with a Slow-Scan CCD-Camera (ProScan, 1024×1024, Scheuring, Germany) with ITEM-Software (Olympus Soft Imaging Solutions, Münster, Germany). Contrast and brightness were adjusted with Adobe Photoshop CS3.

### Phylogenomic analysis

The phage dataset comprised of 24 genomes which were compared pairwise on the proteome level using the Genome Blast Distance Phylogeny approach (GBDP) [57-59]. During distance calculation GBDP considers and corrects for several factors that could potentially bias the results such as overlapping HSPs. In general, GBDP was shown to yield robust results even in the presence of a significant amount of paralogous genes, large repeats and partially incomplete genomes. Further, GBDP does not use arbitrary selections of marker genes but rather detects all available matches (i.e., high-scoring segment pairs, HSPs) between pairs of genomes (e.g., via BLAST + [62]). The resulting set of pairwise distances was subsequently used to infer a phylogenetic tree under the balanced minimum evolution criterion [60] as shown previously [74]. If distance values could not be obtained due to the lack of HSPs between the respective proteomes, twice the maximum distance observed in the matrix was used as a substitute (as in [75]). Since GBDP is capable of calculating a set of bootstrap replicates for each pairwise distance [59], the inferred tree was augmented with branch support values to assess its statistical significance.

## Competing interests

The authors declare that they have no competing interests.

## Authors` contributions

JW, BD and CR designed experiments; JW and BD performed experiments; JPMK conducted phylogenomic analysis; MR performed morphological analysis via transmission electron microscopy, JW and BB did sequence analysis, JW wrote manuscript. All authors read and approved the final manuscript.
